# Adherence to Accelerometer Use in Older Adults Undergoing mHealth Cardiac Rehabilitation: Secondary Analysis of a Randomized Clinical Trial

**DOI:** 10.2196/80522

**Published:** 2025-12-23

**Authors:** Souptik Barua, Dhairya Upadhyay, Stephanie Pena, Riley McConnell, Ashwini Varghese, Samrachana Adhikari, Erik LeRoy, Antoinette Schoenthaler, John A Dodson

**Affiliations:** 1 Division of Precision Medicine Department of Medicine New York University Grossman School of Medicine New York, NY United States; 2 Leon H Charney Division of Cardiology Department of Medicine New York University Grossman School of Medicine New York, NY United States; 3 Department of Population Health New York University Grossman School of Medicine New York, NY United States; 4 Rusk Department of Rehabilitation Medicine New York University Grossman School of Medicine New York, NY United States

**Keywords:** cardiac rehabilitation, mHealth, mobile health, wearable, accelerometry, artificial intelligence, AI, phenotyping, physical activity, Fitbit

## Abstract

**Background:**

Wearable accelerometers, which continuously record physical activity metrics, are commonly used in mobile health–enabled cardiac rehabilitation (mHealth-CR). The association between adherence to accelerometer use during mHealth-CR and improvement in clinical outcomes, such as functional capacity, is understudied. The emergence of artificial intelligence (AI) technology provides novel opportunities to investigate accelerometry use patterns in relation to mHealth-CR outcomes.

**Objective:**

In this study, we sought to use an AI clustering framework to identify distinct behavioral phenotypes of adherence to accelerometer use. We then aimed to quantify the association of these adherence phenotypes with functional capacity improvements in older adults undergoing mHealth-CR.

**Methods:**

We analyzed data from the RESILIENT (Rehabilitation at Home Using Mobile Health in Older Adults After Hospitalization for Ischemic Heart Disease) trial, the largest randomized clinical study to date comparing mHealth-CR versus usual care in older adults (aged ≥65 years). Intervention arm participants were instructed to wear a Fitbit accelerometer for the 3-month study duration. Adherence to accelerometer use was quantified as overall adherence (percentage of days worn) via k-means clustering AI-derived measures and compared with changes in 6-minute walk distance (6-MWD), adjusted for demographic and clinical covariates.

**Results:**

Among 271 participants with a mean age of 71 years (SD 8), of whom 198 (73%) were male, accelerometers were worn for an average of 76 days (95% confidence limits 73,78) over 3 months. Adjusted analyses showed a weak association between days of wear and improvement in 6-MWD, with every 30 additional days associated with an 11-meter improvement (*P*=.08). Our k-means clustering framework identified adherence phenotypes at two resolutions: low resolution (k=2 clusters) and high resolution (k=8 clusters). The consistently high adherence cluster trended toward a 24.6-meter improvement in 6-MWD compared to the low and declining adherence clusters (n=39; 95% CI 0.7-49.9; *P=.*06). The 8-cluster phenotyping revealed a richer set of adherence patterns, with the consistently high adherence cluster in this analysis having a 38.5-meter (95% CI 2.2-74.7; *P*=.04) improvement in 6-MWD than the low adherence cluster, as well as greater average daily steps over the 3-month intervention (mean 7518, SD 3415 vs mean 4800, SD 2920 steps; *P*=.008).

**Conclusions:**

A time-series AI clustering framework identified a range of behavioral phenotypes representing different degrees of adherence to accelerometer use. Regression analysis identified a weak association between the higher adherence phenotype and functional capacity improvement in older adults undergoing mHealth-CR. Our AI-derived accelerometry adherence phenotypes may offer a new approach to tailor mHealth-CR regimens to individual patients, potentially leading to better outcomes in this high-risk population.

**Trial Registration:**

ClinicalTrials.gov NCT03978130; https://clinicaltrials.gov/study/NCT03978130

**International Registered Report Identifier (IRRID):**

RR2-10.2196/32163

## Introduction

Mobile health–enabled cardiac rehabilitation (mHealth-CR) uses portable electronic devices to deliver remote exercise and counseling services for patients with cardiovascular disease, including ischemic heart disease and heart failure [1,2]. Wearable accelerometers have become an integral component in mHealth-CR regimens, capturing an array of data on parameters including step count and intensity of activity [3,4], which provide regular feedback to participants and exercise therapists providing counseling. A recent review supported both the safety and effectiveness of mHealth-CR [5], including several studies that incorporated accelerometers [6-8].

Multiple randomized controlled trials have demonstrated that CR participants who wore accelerometers on average had a significantly greater increase in daily step counts compared with those who received usual care [9-11]. The use of accelerometers has also been associated with improvements (albeit moderate) in other relevant clinical outcomes, such as functional capacity [12-14]. While it has been speculated that observed improvements may be modulated by adherence or consistency of accelerometer use during mHealth-CR interventions, to date, there are limited objective data on this association [15,16]. There is also little evidence on how distinct patterns of adherence behavior (eg, high adherence throughout versus declining adherence) correlate with changes in functional capacity.

In response to these gaps in knowledge, we analyzed accelerometry data from the RESILIENT (Rehabilitation at Home Using Mobile Health in Older Adults After Hospitalization for Ischemic Heart Disease) study [17] to examine how adherence to accelerometer use impacted outcomes. Though still limited, there is a growing use of artificial intelligence (AI) and machine learning in CR, focused primarily on personalizing CR goals [18,19] and predicting outcomes [20]. In this study, we used time-series AI algorithms to identify distinct phenotypes of accelerometer adherence behavior and quantify clinical improvements for each phenotype. This may offer opportunities to tailor mHealth-CR regimens to specific behavioral phenotypes, leading to higher adherence to physical activity recommendations and ultimately improved clinical outcomes among older adults undergoing mHealth-CR.

## Methods

### Study Population

RESILIENT (NCT03978130) was a randomized clinical trial investigating the effectiveness of mHealth-CR in older adults with ischemic heart disease [17]. The trial was conducted at 5 academic hospitals within 4 health systems: New York University (NYU) Langone Health Main Campus (New York, NY), NYU Langone Health Long Island (Mineola, NY), Bellevue Hospital (New York, NY), University of Massachusetts (Worcester, MA), and Yale-New Haven Health (New Haven, CT). Enrollment occurred between on January 9, 2020, and January 10, 2024, with the last follow-up visit on April 22, 2024. The trial enrolled 400 participants and compared mHealth-CR (n=298) to usual care (n=102). The study participants were aged 65 years or older, had a hospital visit for ischemic heart disease (acute myocardial infarction or elective coronary revascularization), and did not have a diagnosis of dementia or other neurodegenerative disorder. Detailed inclusion and exclusion criteria have been described previously [21] but are also provided here.

### Inclusion Criteria

The inclusion criteria were (1) age 65 years or older and (2) a hospital visit for acute myocardial infarction or coronary revascularization procedures (including percutaneous coronary intervention or coronary artery bypass graft surgery).

### Exclusion Criteria

The exclusion criteria were (1) dependence on walkers for mobility or inability to ambulate; (2) moderate to severe cognitive impairment that impacts daily activities; (3) refusal or inability to provide informed consent; (4) presence of a percutaneous coronary intervention–related groin hematoma impeding brisk walking; (5) current incarceration; (6) inability to use mobile health software in English or Spanish; (7) severe osteoarthritis or recent joint replacement (within the last 3 months); (8) progressive movement disorders, such as Parkinson disease; (9) life expectancy estimated to be less than 3 months; (10) clinical concerns about safety or nonadherence identified by health care professionals; and (11) adverse reaction during a screening 6-minute walk test (eg, significant drop in systolic blood pressure ≥15 mm Hg, chest pain, or ventricular arrhythmia).

In this study, we focused on the participants in the intervention arm, all of whom were provided with Fitbit Inspire devices (Fitbit LLC). The study was approved using a single Institutional Review Board mechanism administered through the NYU Grossman School of Medicine.

### Trial Intervention

Details of the intervention have been published previously [21]. Briefly, participants in the intervention group were enrolled in a 3-month mHealth-CR program, mirroring the duration of traditional ambulatory CR programs. This program comprised 3 integrated components: mHealth-CR software, counseling by an exercise therapist, and remote physiological monitoring. The mHealth-CR software, developed by Moving Analytics, was provided on a Samsung Galaxy tablet (Samsung Electronics Co, Ltd) for the duration of the trial. Counseling involved an initial in-person visit and weekly phone sessions, with participants instructed to exercise at least 5 days a week. The intervention adhered to the United States Physical Activity Guidelines for Americans, second edition [22], which is endorsed by the American Heart Association. Specifically, participants were recommended to aim for at least 150 minutes per week of moderate-intensity aerobic physical activity, spread throughout the week (eg, 5 sessions of 30 minutes each), muscle-strengthening activities at least 2 days per week (using provided elastic resistance bands), and balance training, as recommended for older adults [21]. Exercise intensity was rated using the Borg Rating of Perceived Exertion (target range 11-14), and recommendations were adjusted based on self-reported exertion and step count data during weekly calls in line with the principle that lower exercise targets are adapted as endorsed by the guidelines. Remote monitoring was conducted using a Fitbit Inspire accelerometer and an Omron HEM-920T blood pressure cuff, both connected via Bluetooth to the study tablet, with data accessible through the Moving Analytics platform. This study focused on the analysis of the accelerometry data, which is detailed in the subsequent sections.

### Accelerometry Data

#### Data Extraction

Participants assigned to the intervention arm were instructed to wear a Fitbit Inspire device continuously for a period of 90 days as a component of the mHealth-CR program. The first 50% of the participants wore the Fitbit Inspire, while the last 50% of participants wore the Fitbit Inspire 2, following the former’s discontinuation. All Inspire models use the same activity assessment algorithms. Upon completion of the intervention period, participant-level data were exported from the Fitbit website in JSON format for each study participant. After downloading the JSON files from the Fitbit website, we used a Python script to extract and aggregate daily activity metrics, including steps, distance, and active minutes (ie, sedentary, lightly active, fairly or moderately active, and very or vigorously active) for each participant. Daily minutes of moderate-to-vigorous physical activity (MVPA) were computed by adding moderate and vigorous activity minutes for each day. The data were parsed, time stamps were standardized to local dates, and the metrics were summed by date. The data set was subset to include only the dates corresponding to the 90-day intervention period for each participant. This process resulted in a consolidated 90-day data set of daily step count, sedentary time, and minutes of MVPA for each participant, which was exported to Excel (Microsoft Corp) format for subsequent analysis.

#### Defining Overall Adherence to Accelerometer Use

The first measure of adherence we computed was the overall adherence, defined as the percentage of days out of the 90-day measurement period the participant wore the accelerometer. We determined a “valid wear day” as any day on which the participant recorded at least 100 steps on their Fitbit. This threshold was established based on prior research indicating that fewer than 100 steps per day likely reflects non–wear time [23]. Any given day in the 90-day intervention period was assigned a binary value (0/1) depending on whether the day was a valid (1) or invalid (0) wear day. The overall adherence was computed as the number (and percentage) of valid days out of the 90 days.

#### Accelerometer Adherence Trajectories

Going beyond the simple overall adherence measure above, we next sought to quantify how adherence evolved during the 90-day measurement period. To do this, the 90-day zero or one sequence of valid wear days was divided into six 15-day periods to summarize adherence patterns efficiently and robustly over the course of the study. Adherence for each of the six 15-day periods (days 1-15, 16-30, 31-45, 46-60, 61-75, and 76-90) was defined as the number of valid wear days in each 15-day window. Finally, we defined the “adherence trajectory” for each participant as the time series of 6 numbers representing the adherence over each successive 15-day window.

#### AI-Derived Adherence Phenotypes

We then used the k-means clustering technique to identify distinct accelerometry adherence phenotypes based on the shape of participants’ adherence trajectories. The well-known silhouette index was used to identify the optimal number *k* of clusters (or phenotypes). Based on the silhouette curve (Figure S1 in Multimedia Appendix 1), 3 candidates for *k* were identified as k=2, 5, or 8, providing a low, intermediate, and high-resolution view into participants’ adherence patterns, respectively. We focused on the low- and high-resolution views in this study to avoid redundancy while providing a full picture of how adherence to accelerometry usage correlated with our outcome of interest.

#### Computing Activity Metrics Per Phenotype

Finally, to examine whether higher adherence phenotypes also had higher activity levels overall, we computed the average daily step count, daily sedentary time, and weekly minutes of MVPA over the 90-day study period for each phenotype.

### Outcome

Our study outcome was the change in 6-minute walk distance (6-MWD) from baseline to the end of the study (3 months), which was also the primary outcome of the RESILIENT efficacy analysis published previously [17]. The 6-MWD assesses aerobic capacity and endurance by measuring the distance walked on a flat surface within 6 minutes using a standardized course. The test was performed by a clinically credentialed assessor (nurse or exercise therapist) at baseline and at the end of the 3-month intervention. The difference in distance walked (in meters) between baseline and 90 days served as the primary measure to evaluate the intervention's effect on functional capacity.

### Covariates

We included several variables as covariates in our linear regression models, including age, BMI, self-reported sex, race, ethnicity, and site of enrollment. We included a composite comorbidity score as a covariate by assigning binary values (1 = presence, 0 = absence) to diabetes, heart failure, chronic kidney disease, osteoarthritis, and depression (Patient Health Questionnaire-8 score ≥10), abstracted from each patient’s medical record. These were summed to generate a total score ranging from 0 to 5 for each participant, which was used in our regression models to adjust for comorbid conditions.

### Statistical Analysis

Pairwise comparisons were performed using a *t* test for continuous-valued variables and a chi-square test for categorical variables. Linear regression was used to model the association between adherence metrics as the exposure (either overall adherence or adherence phenotype) and change in 6-MWD from baseline to the end of the intervention (ie, 90 days as the outcome), adjusted for the covariates noted previously. A comparison of physical activity metrics for the adherence phenotyping was done using an analysis of variance (ANOVA) followed by post hoc pairwise comparison using the Tukey's honestly significant difference test. A *P* value of .05 was set a priori as the criterion for statistical significance. All analyses were performed with Python software using the “statsmodels” package [24].

### Handling Missing Data

#### Missing Accelerometry Data

For the adherence phenotyping analysis, days of missing accelerometry data were treated as nonvalid wear days and directly informed the phenotyping analysis. In contrast, for the estimation of 90-day step counts, sedentary time, and MVPA for each phenotype, missing values of these measures were imputed as follows: To estimate step counts over each 15-day window with missing data from invalid wear days, we implemented an imputation strategy using a rolling window approach. Days were grouped into consecutive 15-day intervals. Within each interval, missing steps, sedentary time, and MVPA values for invalid days were imputed by calculating the mean of the valid days within the same interval. If an interval contained no valid days, the mean values from the most recent previous interval with valid data were used for imputation. This method was chosen to preserve the temporal structure of the data and to minimize potential biases associated with missing data.

#### Missing 6-MWD Data

Only participants who completed the 6-MWD at both baseline and after 3 months were included in the analysis. The characteristics of the participants who were excluded from the analysis were compared to the final included sample to assess if our findings would generalize to the subset that was excluded.

Ethical Considerations This study followed a single institutional review board process and received approval from the NYU Grossman School of Medicine Institutional Review Board (18-02017) [17]. Participants provided written informed consent, including for the physical activity data analysis from Fitbit. All data analyzed in this study were de-identified. Participants were compensated a maximum of US $115, including $25 for completing the baseline visit and $90 for completing the follow-up visit; there was no specific compensation tied to adherence to accelerometer wear.

## Results

### Participant Characteristics

A total of 298 participants were enrolled in the intervention arm of the study between January 9, 2020, and January 10, 2024. Among them, 27 participants were missing 6-MWD at 3 months due to dropout or loss to follow-up, and they were excluded from our analysis. The final sample of 271 participants was a mean of 71 (SD 8) years old, 198 (73%) male, 55 (20%) non-White, and 21 (8%) Hispanic/Latinx. The mean BMI was 28 (SD 5) kg/m², and 165 (61%) participants had at least 1 comorbidity. At baseline, the mean 6-MWD was 396.8 (SD 95.1) meters. After the 3-month intervention period, this increased to 435.8 (SD 101.4) meters (*P*<.001) (Table 1).

While they were a small fraction of the enrolled cohort, we compared the characteristics of the 27 excluded participants with the 271 included in the final analysis (Table S1 in Multimedia Appendix 1). These participants were slightly older (mean 75, SD 7 years), more racially diverse (13/27 or 48% non-White), and had a lower baseline 6-MWD (mean 309.8, SD 88.4 meters) (all *P*<.05).

**Table 1:** Cohort characteristics (N=271).

Variable			Value
Age (years), mean (SD)			71 (8)
Male sex, n (%)			198 (73)
**Race, n (%)**			
	White	216 (80)	
	Black or African American	21 (8)	
	Asian	14 (5)	
	Other	15 (6)	
	Missing/unknown	5 (2)	
**Ethnicity, n (%)**			
	Not Hispanic or Latinx	249 (92)	
	Hispanic or Latinx	21 (8)	
	Missing/unknown	1 (0.4)	
BMI (kg/m^2^), mean (SD)			27.8 (4.7)
**Comorbidity score (1-5), n (%)**			
	0	106 (39)	
	1	115 (42)	
	2+	48 (18)	
	Missing/unknown	2 (0.7)	
**Sites, n (%)**			
	NYU^a^ Langone	140 (52)	
	UMass^b^	74 (27)	
	Winthrop	39 (14)	
	Bellevue and Yale	18 (7%)	
**6-MWD^c^** **(meters), mean (SD)**			
	Baseline	396.8 (95.1)	
	3 months	435.8 (101.4)	

^a^NYU: New York University.

^b^UMass: University of Massachusetts.

^c^6-MWD: 6-minute walk distance.

### Overall Adherence and Change in 6-MWD

**Figure 1:**
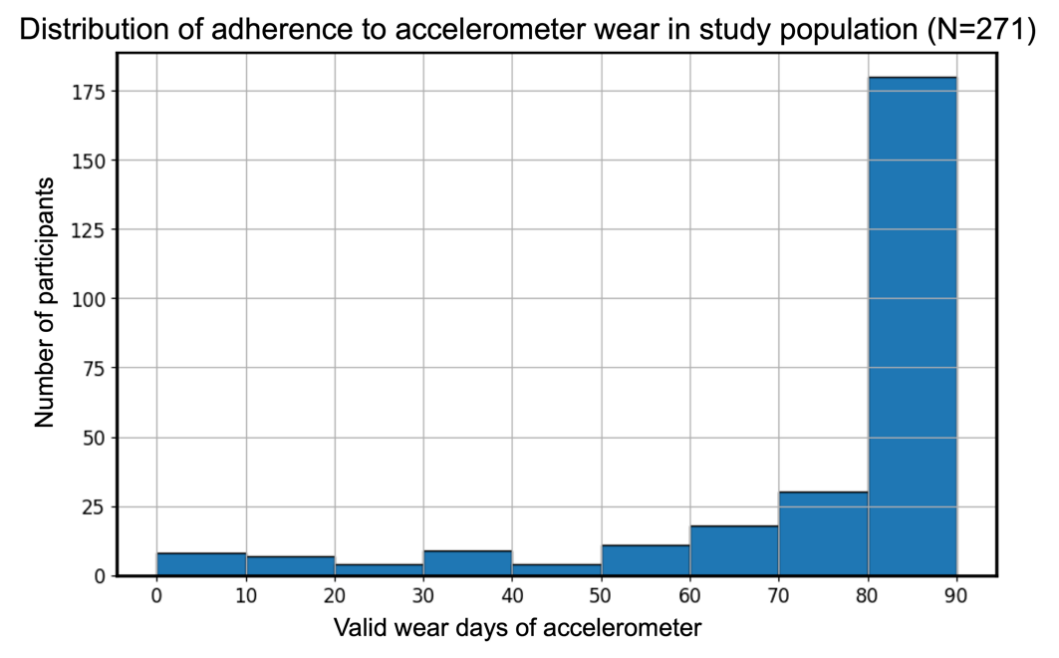
Histogram showing the distribution of adherence to accelerometer use, defined as number of valid days of accelerometer wear in the 90-day measurement period. The X-axis shows the number of valid wear days, and the Y-axis shows the number of participants with the corresponding number of valid accelerometer wear days (N=271).

The average overall adherence to accelerometer use was 84% (95% CI 81%-87%) or 76 (95% CI 73-78) valid wear days over the 3-month measurement period. The distribution of the overall adherence is shown in Figure 1. Among the 271 participants, 252 (93%) wore the accelerometer for at least 30 days, and 228 (84%) wore it for at least 60 days out of the 90-day measurement period.

We first examined the univariate association between overall adherence and change in 6-MWD and found a positive association between the two (β=0.44; 95% CI 0.06-0.82; *P*=.02). After adjusting for demographic and clinical covariates, the overall adherence had a nonsignificant association with change in 6-MWD (β=0.37; 95% CI –0.04 to 0.77; *P*=.08) (Table 2). These findings indicate an 11-meter improvement in 6-MWD for every additional 30 days of adherence to accelerometer use. Age had a weak association with change in 6-MWD, with older participants having greater improvement in their 6-MWD scores (β=1.09; 95% CI: –0.02 to 2.21; *P*=.05). None of the other variables had a significant association with change in 6-MWD.

**Table 2:** Linear regression model with overall accelerometer adherence as the exposure and change in 6-minute walk distance (6-MWD) as the outcome, adjusted for demographic and clinical covariates (N=271).

Variable		Coefficient estimate (95% CI)	*P* value
Intercept		–63.58 (–170.71 to 43.54)	.24
Age		1.09 (–0.02 to 2.21)	.055
Female sex (reference: male)		6.20 (–13.69 to 26.08)	.54
Non-White race (reference: White)		–16.13 (–40.85 to 8.59)	.20
**Ethnicity (reference: Not Hispanic or Latino)**			
	Hispanic or Latino	5.42 (–31.63 to 42.47)	.77
	Not reported	–6.82 (–147.22 to 133.58)	.92
BMI		–0.08 (–2 to 1.84)	.94
**Study site (reference: NYU^a^** **Langone)**			
	Bellevue/Yale	–10 (–48.90 to 28.91)	.61
	UMass^b^	6.04 (–15.26 to 27.35)	.58
	Winthrop	–7.64 (–33.92 to 18.65)	.57
Comorbidity Score		0.81 (–9.68 to 11.30)	.88
Overall accelerometry adherence		0.37 (–0.04 to 0.77)	.08

^a^NYU: New York University.

^b^UMass: University of Massachusetts.

### Shape of Adherence Trajectories and Association With Changes in 6-MWD

#### Explanation

Going beyond the overall adherence for the whole 90-day period, we subsequently examined the association of fine-grained AI-derived adherence phenotypes and change in 6-MWD. Following the rationale provided in the Methods section, we present our results for adherence phenotypes derived using the k-means clustering algorithm at 2 resolutions: low-resolution (k=2) and high-resolution (k=8).

#### K-Means Using 2 Clusters

**Figure 2:**
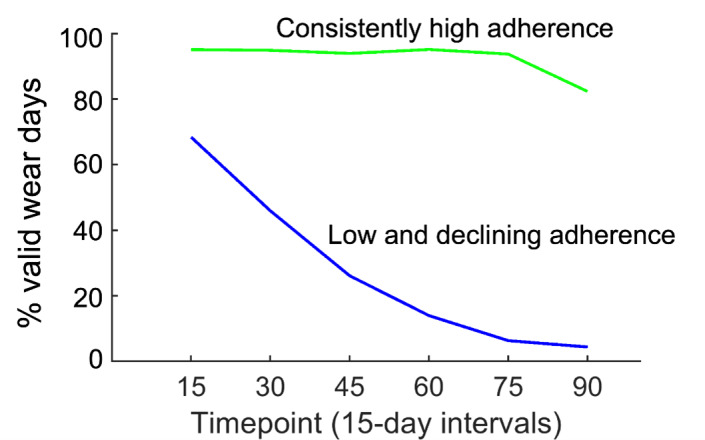
Accelerometry adherence phenotypes identified using k-means clustering (k=2). The 2 trajectories represent the average of all adherence trajectories within each of the two clusters. The X-axis represents a 15-day time point (days 1-15; 16-30,…, 76-90). The Y-axis represents adherence for each 15-day window as the percentage of days that are valid wear days.

The accelerometry adherence phenotypes derived using k-means with k=2 are shown in Figure 2. The 2 clusters identified represent a consistently high adherence (mean 83, SD 9 valid wear days) phenotype and a low and declining adherence (mean 25, SD 17 valid wear days) phenotype. The low adherence cluster compared to the consistently high cluster was more racially diverse (44% vs 16% non-White; *P*<.001) and more likely to be from a non-NYU Langone study site (50% vs 36% non-NYU Langone; *P*=.007) (Table S2 in Multimedia Appendix 1). The consistently high adherence cluster had an improvement in 6-MWD from a mean of 400.3 (SD 97.8) meters at baseline to 443.8 (SD 99.5) meters at 3 months (*P*<.001). In contrast, participants in the low and declining adherence cluster showed no statistically meaningful change in 6-MWD, increasing from a mean of 373.7 (SD 73.4) meters at baseline to 388.5 (SD 100.6) meters at 3 months (*P*=.24). In the adjusted analysis, adherence phenotype had a weak association with change in 6-MWD, with participants in the low and declining adherence cluster having on average 25 meters less improvement in 6-MWD compared to those in the consistently high-adherence cluster (β=–24.6; 95% CI: –49.9 to 0.7; *P*=.06) (Table 3). Older age had a weak association with change in 6-MWD (*P*=.054).

**Table 3:** Linear regression model with k-means clustering (k=2) accelerometer adherence phenotype as the exposure and change in 6-minute walk distance (6-MWD) as the outcome, adjusted for demographic and clinical covariates (N=271).

Variable			Coefficient estimate (95% CI)		*P* value
Intercept			–30.64 (–132.01 to 70.74)		.55
Age			1.10 (–0.02 to 2.21)		.054
Female sex (reference: male)			6.70 (–13.16 to 26.55)		.51
Non-White race (reference: White)			–15.53 (–40.28 to 9.22)		.22
**Ethnicity (reference: Not Hispanic or Latino)**					
	Hispanic or Latino	5.39 (–31.63 to 42.40)		.77	
	Not reported	–5.60 (–145.83 to 134.64)		.94	
BMI			–0.15 (–2.07 to 1.77)		.88
**Study site (reference: NYU^a^** **Langone)**					
	Bellevue/Yale	–10.46 (–49.30 to 28.37)		.60	
	UMass^b^	6.29 (–14.95 to 27.54)		.56	
	Winthrop	–7.77 (–34.04 to 18.50)		.56	
Comorbidity Score			0.25 (–10.14 to 10.63)		.96
**Accelerometry adherence phenotype (reference: consistently high adherence)**					
	Low and declining adherence	–24.57 (–49.88 to 0.73)		.06	

^a^NYU: New York University.

^b^UMass: University of Massachusetts.

#### K-Means Using 8 Clusters

**Figure 3:**
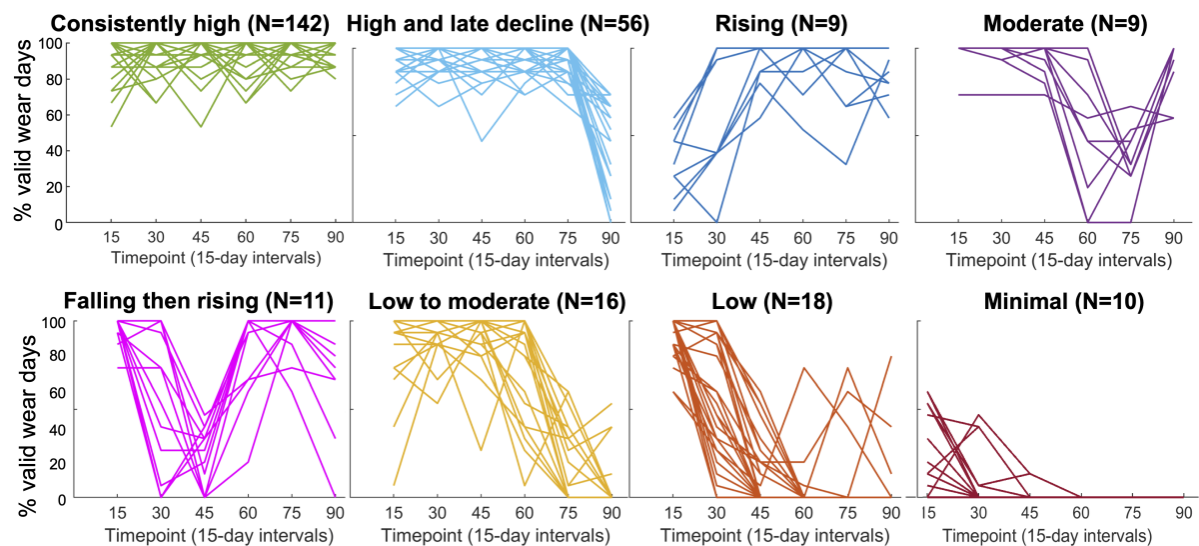
Accelerometry adherence phenotypes identified using k-means clustering (k=8). Each trajectory represents a participant. The X-axis represents a 15-day time point (days 1-15; 16-30,…, 76-90). The Y-axis represents adherence for each 15-day window as the percentage of days that are valid wear days.

The accelerometry adherence phenotypes derived using k-means with k=8 are shown in Figure 3, illustrating more fine-grained adherence behavior patterns in our participants. For each cluster, we labeled them based on their visual appearance as follows: consistently high adherence (n=142, 98% overall adherence), high with late decline adherence (n=56, 89% overall adherence), rising adherence (n=9, 74% overall adherence), moderate adherence (n=9, 77% overall adherence), falling then rising adherence (n=11, 72% overall adherence), low-to-moderate adherence (n=16, 64% overall adherence), low adherence (n=18, 29% overall adherence), and minimal adherence (n=10, 7% overall adherence). The rising, moderate, and falling then rising adherence clusters had overall adherence levels within 10 percentage points but had different adherence trajectory shapes, indicating differences in accelerometry use not captured by the overall adherence metric (Figure 3).

#### Descriptive Statistics for K=8 Clusters

When examining the distribution of participants within each cluster (Table S3 in Multimedia Appendix 1), over two-thirds of our participants were placed in the consistently high adherence cluster (142/271, 52%) or the high with late decline adherence cluster (56/271, 21%), indicating a relatively high adherence to accelerometry use over the 3-month study period. We then examined the baseline, 3-month, and change in 6-MWD over 3 months for each cluster (Table S3 in Multimedia Appendix 1). The participants in the consistently high, high with late decline, and rising adherence clusters had a significant improvement in their 6-MWD of 46 meters (*P<.*001), 36 meters (*P*=.001), and 87 meters (*P*<.001), respectively. Participants in the other clusters did not have a statistically meaningful change in their 6-MWD over the 3-month intervention. A notable exception was the minimal adherence cluster, which had the least overall adherence but a borderline significant improvement in 6-MWD (*P=.*05). Detailed demographic and clinical characteristics for each of the 8 clusters are also provided in Table S4 in Multimedia Appendix 1.

#### Association of K=8 Adherence Phenotypes With Changes in 6-MWD

Next, we aimed to estimate the association between the adherence phenotypes for the k=8 k-means clusters and changes in 6-MWD, adjusted for covariates (Table 4). Relative to the consistently high adherence cluster, the low adherence cluster had a 39-meter lower change in 6-MWD (β=–38.47 (95% CI –74.73 to –2.21; *P*=.04). No other demographic or clinical covariate was associated with the outcome in this regression analysis. Of note, the minimal adherence cluster no longer showed an association with change in 6-MWD in the adjusted analysis.

**Table 4:** Linear regression model with k-means clustering (k=8) accelerometer adherence phenotype as the exposure and change in 6-minute walk distance (6-MWD) as the outcome, adjusted for demographic and clinical covariates. The clusters are ordered from the greatest to the least overall adherence (N=271).

Variable			Coefficient estimate (95% CI)		*P* value
Intercept			–20.44 (–123.60 to 82.72)		.70
Age			0.89 (–0.24 to 2.03)		.12
Female sex (reference: male)			8.27 (–11.68 to 28.23)		.41
Non-White race (reference: White)			–18.29 (–43.18 to 6.60)		.15
**Ethnicity (reference: Not Hispanic or Latino)**					
	Hispanic or Latino	9.50 (–27.98 to 46.98)		.62	
	Not reported	–8.69 (–148.78 to 131.41)		.90	
BMI			0.08 (–1.87 to 2.02)		.94
**Study site (reference: NYU^a^** **Langone)**					
	Bellevue/Yale	–6.77 (–45.82 to 32.27)		.73	
	UMass^b^	4.70 (–16.80 to 26.19)		.67	
	Winthrop	–9.96 (–36.74 to 16.81)		.46	
Comorbidity Score			0.51 (–10.06 to 11.08)		.92
**Accelerometry adherence phenotypes** **(reference: Consistently high) (N=142)**					
	High and late decline (n=56)	–8.41 (–30.79 to 13.98)		.46	
	Rising (n=9)	38.92 (–11.92 to 89.76)		.13	
	Moderate (n=9)	20.10 (–28.59 to 68.79)		.42	
	Falling then rising (n=11)	–16.85 (–60.49 to 26.78)		.45	
	Low to moderate (n=16)	–27.80 (–66.95 to 11.36)		.16	
	Low (n=18)	–38.47 (–74.73 to –2.21)		.04	
	Minimal (n=10)	–18.46 (–64.51 to 27.60)		.43	

^a^NYU: New York University.

^b^UMass: University of Massachusetts.

#### Steps, Sedentary Time, and MVPA

Finally, for each of the 8 clusters, we computed standard accelerometry–derived physical activity parameters such as steps and time spent sedentary or in MVPA over the whole 3-month intervention period. The accelerometry metrics on days of nonadherence (<100 steps) were imputed using the rolling window mean imputation method noted in the Handling Missing Data subsection in the Methods section. The daily steps, weekly MVPA, and daily sedentary time for each of the 8 phenotypes are shown in Table S5 in Multimedia Appendix 1. There was a statistically significant difference in daily steps (*P*=.008) and sedentary time between the 8 phenotypes (*P*=.005). In post hoc pairwise comparisons, the low adherence phenotype (mean 4800, SD 2920 steps) had 2718 steps fewer (95% CI 149-5286) than the consistently high phenotype (mean 7518, SD 3415 steps; *P*=.03). No pairwise comparisons were significant for weekly MVPA and sedentary time.

## Discussion

### Principal Findings

Wearable accelerometers have become an integral component of mHealth-CR interventions [25]. Using accelerometry data from the RESILIENT trial, we sought to examine whether adherence to accelerometer use was associated with improved functional capacity in older adults undergoing mHealth-CR. There were several key findings. First, we observed that adherence to accelerometry use was reasonably high: 228 (84%) out of 271 participants wore the accelerometer for at least 60 days out of the 90-day measurement period. Second, we found that a simple overall adherence metric—the percentage of valid wear days over the 90-day intervention—had a weak association with improvement in 6-MWD in the adjusted analysis. Third, using an AI-based k-means clustering algorithm to differentiate accelerometer use patterns with more granularity, we observed that higher adherence phenotypes were weakly associated with greater improvements in 6-MWD compared to lower adherence phenotypes. The lack of statistical significance may be explained by the smaller sample size of lower adherence phenotypes, as fewer than 20% of participants were assigned to the lower adherence phenotypes at either clustering resolution (k=2 or 8). Previous studies have found improvements in 6-MWD of 25 to 35 m as clinically meaningful [26-28]. Notably, in both the k=2 and k=8 clustering analysis, the higher adherence phenotypes tended to have above 40 meters of improvement, while the lower adherence phenotypes showed lower than 25 meters of improvement in 6-MWD on average over 3 months, showing a potential relationship between accelerometry use adherence and functional capacity improvement. However, some of the higher adherence clusters that showed significant improvements in 6-MWD in the unadjusted analysis were likely biased due to their small sample sizes; therefore, further research is needed to examine their generalizability. While underpowered to detect differences in improvement in functional capacity, our AI phenotyping still provided a rich view into behavioral patterns associated with accelerometry use over the course of the intervention. AI-derived behavioral phenotypes may be particularly amenable to personalized mHealth-CR that leverage behavioral economic principles to increase engagement and adherence [29].

### Comparison to Prior Work

The relatively high adherence to accelerometer use among older adults in our study mirrors another study examining adherence to use of a wrist accelerometer in 303 adults from the SafeHeart study, which found that 4 in 5 participants wore their accelerometer for more than 75% of the study period of 6 months [30]. Other studies in older adults also found high adherence to mHealth-CR [2,31]. More broadly, there is increasing evidence of the feasibility of home-based exercise interventions using wearables and mHealth in older adults [32,33]. However, these findings and our own must be interpreted in the context of a clinical research study, where participants are more highly motivated than the general population; other studies of US adults more generally have found that fewer than half who owned a wearable device reported wearing it daily [34].

### Limitations

Our study has several important limitations. First, we used a convenience sample of intervention arm participants, and the smaller participant counts in lower adherence phenotypes may have prevented us from seeing a stronger association between accelerometry adherence and improvement in 6-MWD. Testing multiple adherence models (overall adherence, k=2 clusters, and k=8 clusters) may have increased the likelihood of a type 1 error. Therefore, the statistically significant findings from the granular 8-cluster analysis need to be interpreted with caution due to the small sample sizes of the lower adherence clusters. In contrast, the minimal adherence cluster of 10 participants showed a borderline significant trend toward improved 6-MWD, contrary to our expectations. While this association disappeared in the adjusted analysis, further investigation in the future may still be important to understand populations who may be engaged with their CR via traditional methods but may not prefer to use a wearable device. Second, the 6-minute walk test can have a ceiling effect [35] and may not show significant improvements in a population with relatively high functional capacity or minimal ambulation issues. A more strenuous test, like a maximal graded exercise test [36], might have revealed larger improvements in our population, although this remains speculative. Third, prior studies have shown that factors such as the type and speed of movement, as well as the placement of the Fitbit, affect the accuracy of step counts [37], which may have influenced the adherence values we calculated. Fitbit devices are also known to have a degree of error due to improper fit [38]. The 100-step threshold for valid Fitbit wear, while based on prior research [23], may fail to account for highly sedentary days where the device was worn. While the estimates of steps, sedentary time, and MVPA using the rolling window mean imputation approach are expected to be robust for 6 of the 8 clusters (>64% adherence; 90% of sample), they were likely biased for the *low* and *minimal* adherence clusters (<30% adherence; 10% of sample). Future studies using more rigorous methods for missing not-at-random data, such as Multiple Imputation by Chained Equations [39] and autoencoders [40], may better estimate these activity metrics in highly nonadherent participants. Only 27 (<10%) out of 298 enrolled participants were excluded from the analysis due to reasons such as dropout or loss-to-follow-up, and this small number is unlikely to alter our study findings. However, we observed this group to be more racially diverse and with a lower baseline 6-MWD than our study sample, which warrants further investigation in future mHealth-CR studies. Finally, our study was performed in older adults (65+ years) with ischemic heart disease; therefore, the accelerometry phenotypes identified in this population may not generalize to mHealth-CR in individuals from younger age groups or with different heart conditions, such as valvular heart disease [41].

### Future Directions

Our use of AI phenotyping demonstrates the ability to identify critical temporal windows for targeted intervention, which may be missed by simpler measures of adherence. For instance, in the 8-cluster analysis, the low adherence phenotype, which had significantly lower improvement in 6-MWD compared to the consistently high adherence phenotype, showed steep drops in Fitbit use in the 30-to-60-day window. Another example is the moderate adherence phenotype, which exhibited a notable decline in adherence between 45 and 60 days, suggesting that increased monitoring and support after 2 months may be particularly beneficial for these patients to maintain engagement with the study intervention. Considering the negative findings of the main RESILIENT trial, these insights are particularly useful to inform future mHealth-CR interventions whereby participant subgroups may be targeted for more or less attention over the course of the intervention. An example clinical implementation strategy could be 3 months of a standard mHealth-CR to identify a patient’s phenotype, followed by 3 months of personalized mHealth-CR programs where the content and frequency are tailored based on the patient’s phenotype. In settings where no more than 3 months of mHealth-CR is feasible, future studies may explore using the first month for phenotyping, followed by personalized mHealth-CR for the next 2 months to incorporate the benefits of personalization within a fixed 3-month time frame. In an era of growing use of AI and digital health interventions, our findings provide a framework for using wearables and AI in optimizing engagement of mHealth-CR, ultimately leading to improved outcomes for more individuals undergoing CR.

### Conclusions

In summary, in a multicenter study of mHealth-CR in older adults, we comprehensively characterized adherence to accelerometer use as an indication of improvement in functional capacity. Using both simple as well as AI-derived methods, we observed a weak association between accelerometer adherence and improvement in the 6-MWD in our study population. Our findings suggest that accelerometers may provide clinicians with objective insights to personalize mHealth-CR regimens, leading to optimal benefits for older adults undergoing mHealth-CR.

## Appendix

Multimedia Appendix 1 Supplementary tables and figures.

## Abbreviations

6-MWD: 6-minute walk distance
AI: artificial intelligence
CR: cardiac rehabilitation
mHealth: mobile health
MVPA: moderate-to-vigorous physical activity

## Data Availability

Data needed to reproduce the findings of this manuscript are available upon reasonable request. Requests for data should be sent to resilient@nyulangone.org, and requests for code should be sent to souptik.barua@nyulangone.org.
