# Supplementary material for: Adherence to Accelerometer Use in Older Adults Undergoing mHealth Cardiac Rehabilitation: Secondary Analysis of a Randomized Clinical Trial
Source: J Med Internet Res. 2025 Dec 23;27:e80522. doi: 10.2196/80522 (PMC12777647; doi:10.2196/80522)

**Multimedia Appendix:** Supplementary tables and figures

**Table S1**: Characteristics of the participants excluded from the analysis due to lack of 3-month 6-MWD information (N=27). Values reported as mean±SD or counts (%). Test statistic and P-value are derived from a t-test (continuous-valued variables) or chi-square test (categorical variables) comparing the 27 excluded participants with the included sample of 271 participants detailed in Table 1. 6-MWD: 6-minute walk distance. SD: Standard deviation.

| Variable | Value | Test statistic | P-value |
| --- | --- | --- | --- |
| Age (years) | 74.84 ± 6.69 | 2.58 | 0.014 |
| Sex   - Female - Male | 9 (33.3%)  18 (66.7%) | 0.23 | 0.63 |
| Race   - Non-White - White | 13 (48.1%)  14 (51.9%) | 9.29 | 0.002 |
| Ethnicity   - Hispanic or Latino - Not Hispanic or Latino - Not reported | 2 (7.4%)  24 (88.9%)  1 (3.7%) | 4.10 | 0.13 |
| BMI (kg/m^2^) | 26.24 ± 3.17 | -2.26 | 0.03 |
| Study site   - Langone - Bellevue/Yale - UMass - Winthrop | 16 (59.3%)  5 (18.5%)  6 (22.2%)  0 (0.0%) | 10.14 | 0.04 |
| Comorbidity Score   - 0 - 1 - 2+ | \| 11 (40.7%) \| \| --- \| \| 11 (40.7%)  5 (18.5%) \| | 0.30 | 0.99 |
| Baseline 6-MWD (meters) | 309.77 ± 88.46 | -4.83 | <0.001 |

**Table S2**: Demographic and clinical characteristics of each accelerometry adherence phenotype (k=2). (N=271) Test statistic is from a t-test for continuous-valued variables and a chi-squared test for categorical variables.

| Variable | Consistently High | Low and Declining | Test statistic | p-value |
| --- | --- | --- | --- | --- |
| **N** | 232 | 39 |  |  |
| Age (years) | 71 ± 8 | 72 ± 6 | 0.84 | 0.40 |
| Sex |  |  | 0.15 | 0.70 |
| ◼  Female | 61 (26%) | 12 (31%) |  |  |
| ◼  Male | 171 (74%) | 27 (69%) |  |  |
| Race |  |  | 13.65 | <0.001 |
| ◼  Non-White | 38 (16%) | 17 (44%) |  |  |
| ◼  White | 194 (84%) | 22 (56%) |  |  |
| Ethnicity |  |  | 3.86 | 0.15 |
| ◼  Hispanic or Latino | 15 (6%) | 6 (15%) |  |  |
| ◼  Not Hispanic or Latino | 216 (93%) | 33 (85%) |  |  |
| ◼  Not reported | 1 (1%) | 0 |  |  |
| BMI (kg/m^2^) | 27.91 ± 4.56 | 26.88 ± 5.47 | -1.11 | 0.27 |
| Study site |  |  | 14.03 | 0.007 |
| ◼  Langone | 115 (50%) | 25 (64%) |  |  |
| ◼  Bellevue/Yale | 10 (4%) | 6 (15%) |  |  |
| ◼  UMass | 70 (30%) | 4 (10%) |  |  |
| ◼  Winthrop | 35 (15%) | 4 (10%) |  |  |
| Comorbidity Score | 1 ± 1 | 1 ± 1 | 5.00 | 0.29 |
| 6-MWD (meters) |  |  |  |  |
| ◼  Baseline: Mean [SD] | 400.35 ± 97.83 | 373.69 ± 73.41 | -1.99 | 0.051 |
| ◼  3 months: Mean [SD] | 443.80 ± 99.55 | 388.50 ± 100.56 | -3.18 | 0.002 |
| ◼  Change: Mean [SD] | 43.45 ± 68.59 | 14.81 ± 76.69 | -2.19 | 0.03 |

**Table S3**: Baseline, 3-month, and change in 6-MWD for each accelerometry adherence phenotype (k=8). P-values representing statistical significance of change in 6-MWD computed using paired t-tests and adjusted for multiple comparisons using Benjamini-Hochberg criterion. Values for 6-MWD reported as mean [SD]. 6-MWD: 6-minute walk distance. SD: Standard deviation. (N=271)

| Adherence phenotype | Counts  (N,%) | Overall adherence (%) | Baseline 6-MWD (meters) | 3-month 6-MWD (meters) | Change in 6-MWD (meters) | P-value for change in 6-MWD |
| --- | --- | --- | --- | --- | --- | --- |
| Consistently high | 142 (52%) | 98% | 411±88 | 458±88 | 46±71 | <0.001 |
| High with late decline | 56 (21%) | 89% | 386±102 | 422±108 | 36±70 | <0.001 |
| Rising | 9 (3%) | 74% | 360±113 | 447±108 | 87±41 | <0.001 |
| Moderate | 9 (3%) | 77% | 418±67 | 477±101 | 59±79 | 0.06 |
| Falling then rising | 11 (4%) | 72% | 401±122 | 429±114 | 28±61 | 0.16 |
| Low to moderate | 16 (6%) | 64% | 358±122 | 370±128 | 11±38 | 0.25 |
| Low | 18 (7%) | 29% | 346±83 | 350±106 | 4±93 | 0.86 |
| Minimal | 10 (4%) | 7% | 407±49 | 429±41 | 23±32 | 0.05 |

**Table S4**: Demographic and clinical characteristics of each accelerometry adherence phenotype (k=8). (N=271)

| **Variable** | Consistently high | High with late decline | Rising | Moderate | Falling then rising | Low to moderate | Low | Minimal |
| --- | --- | --- | --- | --- | --- | --- | --- | --- |
| **N** | 142 | 56 | 9 | 9 | 11 | 16 | 18 | 10 |
| Age (years) | 72 ± 5 | 70 ± 10 | 74 ± 5 | 72 ± 6 | 73 ± 5 | 66 ± 18 | 73 ± 7 | 73 ± 5 |
| Sex |  |  |  |  |  |  |  |  |
| - Female | 40 (28%) | 13 (23%) | 2 (22%) | 1 (11%) | 3 (27%) | 4 (25%) | 8 (44%) | 2 (20%) |
| - Male | 102 (72%) | 43 (77%) | 7 (78%) | 8 (89%) | 8 (73%) | 12 (75%) | 10 (56%) | 8 (80%) |
| Race |  |  |  |  |  |  |  |  |
| - Non-White | 24 (17%) | 6 (11%) | 1 (11%) | 4 (44%) | 2 (18%) | 6 (38%) | 8 (44%) | 4 (40%) |
| - White | 118 (83%) | 50 (89%) | 8 (89%) | 5 (56%) | 9 (82%) | 10 (62%) | 10 (56%) | 6 (60%) |
| Ethnicity |  |  |  |  |  |  |  |  |
| - Hispanic or Latino | 9 (6%) | 3 (5%) | 0 (0%) | 0 (0%) | 1 (9%) | 4 (25%) | 3 (17%) | 1 (10%) |
| - Not Hispanic or Latino | 132 (93%) | 53 (95%) | 9 (100%) | 9 (100%) | 10 (91%) | 12 (75%) | 15 (83%) | 9 (90%) |
| - Not reported | 1 (1%) | 0 | 0 | 0 | 0 | 0 | 0 | 0 |
| BMI (kg/m^2^) | 27.28 ± 4.47 | 28.98 ± 4.38 | 29.14 ± 4.66 | 26.90 ± 4.36 | 28.36 ± 4.57 | 27.95 ± 5.43 | 28.84 ± 5.97 | 24.53 ± 5.25 |
| Study site |  |  |  |  |  |  |  |  |
| - Langone | 72 (51%) | 22 (39%) | 4 (44%) | 7 (78%) | 5 (45%) | 13 (81%) | 10 (56%) | 7 (70%) |
| - Bellevue/Yale | 8 (5%) | 2 (4%) | 0 (0%) | 0 (0%) | 1 (9%) | 2 (12%) | 4 (22%) | 1 (10%) |
| - UMass | 47 (33%) | 18 (32%) | 2 (22%) | 1 (11%) | 2 (18%) | 1 (6%) | 2 (11%) | 1 (10%) |
| - Winthrop | 15 (11%) | 14 (25%) | 3 (33%) | 1 (11%) | 3 (27%) | 0 (0%) | 2 (11%) | 1 (10%) |
| Comorbidity Score | 0.7 ± 0.8 | 0.9 ± 0.9 | 1.0 ± 0.9 | 1.1 ± 0.9 | 1.1 ± 0.7 | 0.7 ± 1.1 | 1.4 ± 1.1 | 1.1 ± 0.6 |

**Table S5**: Physical activity metrics* from the accelerometer over 3 months for each accelerometry adherence phenotype (k=8). (N=271) MVPA: Moderate to Vigorous Physical Activity. ANOVA: Analysis of Variance.

| Adherence phenotype | Counts (N,%) | Daily steps | Weekly MVPA (mins) | Daily sedentary time (mins) |
| --- | --- | --- | --- | --- |
| Consistently high | 142 (52%) | 7518 ± 3415 | 239 ± 198 | 903 ± 327 |
| High with late decline | 56 (21%) | 6833 ± 3166 | 226 ± 210 | 1027 ± 263 |
| Rising | 9 (3%) | 5908 ± 3161 | 238 ± 180 | 1024 ± 323 |
| Moderate | 9 (3%) | 5566 ± 2278 | 156 ± 131 | 1201 ± 128 |
| Falling then rising | 11 (4%) | 6768 ± 2725 | 180 ± 163 | 985 ± 287 |
| Low to moderate | 16 (6%) | 5390 ± 3061 | 139 ± 85 | 1126 ± 243 |
| Low | 18 (7%) | 4800 ± 2920 | 118 ± 137 | 1050 ± 268 |
| Minimal | 10 (4%) | 5240 ± 5697 | 252 ± 412 | 1057 ± 420 |
| ANOVA results |  |  |  |  |
| F-statistic |  | 2.81 | 1.47 | 2.96 |
| p-value |  | 0.008 | 0.18 | 0.005 |

* A rolling window mean imputation approach (see *Methods*) was used to impute the values of physical activity metrics for days of non-wear. Therefore, values of steps, MVPA, and sedentary time for the lower adherence phenotypes (e.g. the Minimal adherence phenotype) are likely to have higher estimation errors.

**Figure S1**: The silhouette index computation to identify optimal number of clusters ‘k’ for our k-means clustering algorithm. Local maxima at k=2 and 8 provide appropriate choices for k. Higher values of k show a slow monotonic increase in the silhouette index, indicating marginal improvements in adding more clusters.


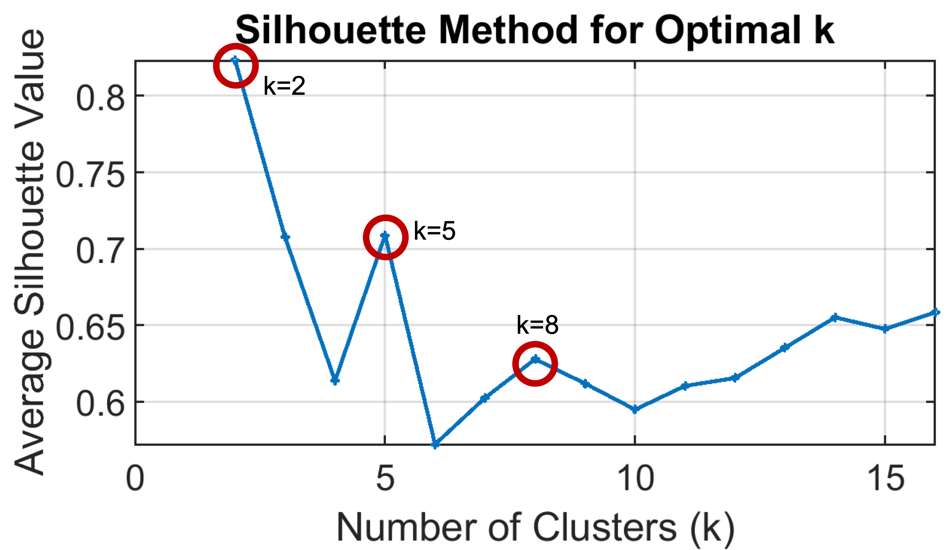

Supplement: Multimedia Appendix 1 [file jmir_v27i1e80522_app1.docx]
